# Epigenetic Regulation of Bone Remodeling and Its Impacts in Osteoporosis

**DOI:** 10.3390/ijms17091446

**Published:** 2016-09-01

**Authors:** Chafik Ghayor, Franz E. Weber

**Affiliations:** 1 Oral Biotechnology & Bioengineering, Center for Dental Medicine, Cranio-Maxillofacial and Oral Surgery, University of Zurich, Zurich 8032, Switzerland; chafik.ghayor@usz.ch; 2 CABMM, Center for Applied Biotechnology and Molecular Medicine, University of Zurich, Zurich 8057, Switzerland; 3 Zurich Center for Integrative Human Physiology (ZIHP), University of Zurich, Zurich 8057, Switzerland

**Keywords:** Genetics–Epigenetics, osteoporosis, bromodomain inhibitor, HDAC inhibitor, bone regeneration

## Abstract

Epigenetics describes mechanisms which control gene expression and cellular processes without changing the DNA sequence. The main mechanisms in epigenetics are DNA methylation in CpG-rich promoters, histone modifications and non-coding RNAs (ncRNAs). DNA methylation modifies the function of the DNA and correlates with gene silencing. Histone modifications including acetylation/deacetylation and phosphorylation act in diverse biological processes such as transcriptional activation/inactivation and DNA repair. Non-coding RNAs play a large part in epigenetic regulation of gene expression in addition to their roles at the transcriptional and post-transcriptional level. Osteoporosis is the most common skeletal disorder, characterized by compromised bone strength and bone micro-architectural deterioration that predisposes the bones to an increased risk of fracture. It is most often caused by an increase in bone resorption that is not sufficiently compensated by a corresponding increase in bone formation. Nowadays it is well accepted that osteoporosis is a multifactorial disorder and there are genetic risk factors for osteoporosis and bone fractures. Here we review emerging evidence that epigenetics contributes to the machinery that can alter DNA structure, gene expression, and cellular differentiation during physiological and pathological bone remodeling.

## 1. Introduction

The human skeleton is a metabolically active organ that undergoes continuous bone remodeling throughout life [1]. The bone remodeling process involves the destruction of mineralized bone followed by the formation of bone matrix that subsequently becomes mineralized. Bone remodeling is critical not only for adapting bone architecture/strength to growth and mechanical needs, but also for repairing microdamage and maintaining calcium homeostasis. The formation and resorption of bone are tightly coupled and this orchestrated balance allows skeletal integrity. However, in certain physiological or pathological conditions, the bone remodeling steps become uncoupled, leading to decreased bone strength and increased fragility [2].

**Figure 1:**
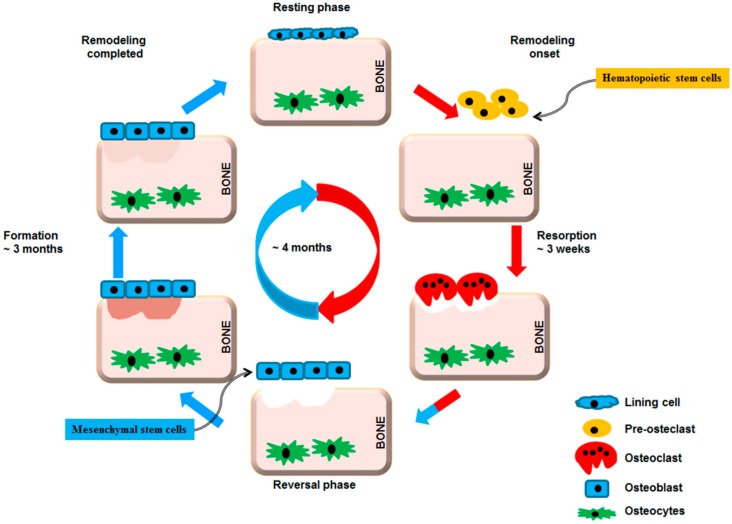
Bone remodeling cycle. A remodeling cycle is initiated by osteoclasts that solubilize bone mineral and degrade the matrix (resorption phase). Osteoclasts originate from hematopoietic stem cells which differentiate first into pre-osteoclast cells which fuse to form multinucleated cells (activated osteoclasts). Monocytes/macrophages remove debris (reversal phase), followed by a bone formation phase performed by osteoblasts, producing osteoid matrix which will mineralize. Growth factors are released from the bone matrix during resorption, which increase the pre-osteoblast population in order to replace damaged bone surfaces.

To maintain homeostasis of bone mass, the bone remodeling cycle involves a series of highly regulated steps that depend on the interaction, differentiation and functions of two cell lineages: the mesenchymal osteoblastic lineage and the hematopoietic osteoclastic lineage (Figure 1).

On a molecular level, the differentiation and activation of bone cells are regulated by a complex signaling network involving both locally secreted factors and systemic hormones. Moreover, it is well established that several environmental and stochastic stressors including diet and chemical exposure can modulate through epigenetic mechanisms both gene expression and lineage decisions [3,4,5]. Thus, the pathological process of multi-factorial bone disorders such as osteoporosis could have an important epigenetic component. Epigenetic factors represent a promising area to link genetics and gene expression with disease risk. A deeper understanding of the molecular and epigenetic regulation of bone cell differentiation and functions will allow us to design and develop new treatments for osteoporosis.

## 2. Epigenetic Regulation of Bone Remodeling

Osteoblasts, bone-forming cells, are derived from mesenchymal stem cells (MSCs). Differentiation of MSCs into specialized cells involves an upregulation of genes involved in differentiation of a specific cell phenotype and downregulation of genes responsible for cell stemness [6]. Key genes for the maintenance of stem cells and their differentiation into particular cell lineages show evidence of epigenetic regulations [7]. These non-genetic alterations are regulated by three main epigenetic modifications: DNA methylation, histone modifications and non-coding RNAs (ncRNAs).

## 3. DNA Methylation

**Figure 2:**
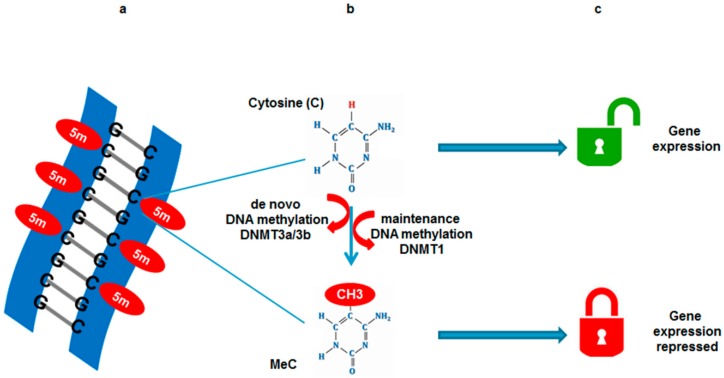
DNA methylation and gene expression. (**a**) DNA methylation occurs at cytosine bases (CpG islands) when a methyl group is added at the 5′ position on the pyrimidine ring by DNA methyl transferases (DNMTs); (**b**) Two types of DNMTs initiate de novo DNA methylation; DNMT3a and DNMT3b to methylate previously unmethylated cytosines (C), whereas maintenance DNMTs (DNMT1) methylate hemi-methylated DNA at the complementary strand; (**c**) Methyl group tags DNA and represses gene expression.

DNA methylation, a well-characterized epigenetic modification, is associated with transcriptional silencing. DNA methylation occurs at the 5′ position of the cytosine ring within CpG dinucleotides and is catalyzed by DNA methyltransferases (DNMTs), including DNMT1, DNMT3a, and DNMT3b [8,9]. DNA methylation is essential for normal development and is associated with gene silencing (Figure 2).

In humans, CpG totals for ~1% and 60%–80% of CpGs are methylated, mainly in heterochromatic regions [10]. Abnormal patterns of DNA methylation influence diseases processes, especially in human tumors [11,12,13,14]. Several studies have suggested that DNA methylation plays an important role in osteoblast differentiation. Promoter methylation changes were observed during the osteogenic differentiation of MSCs. Indeed, in the differentiation of mesenchymal cells towards osteoblastic lineage *runt related transcription factor* *2* (*Runx2*) and *osteocalcin* genes exhibited hypomethylation of the CpGs along with their strong expression [15]. It has been shown that active demethylation of the promoters of *Runx2*, *osteocalcin* and *osterix* genes by growth arrest and DNA-damage-inducible protein (GADD45)-dependent mechanisms is involved in the osteogenic differentiation of adipose-derived MSCs [16]. Interestingly, a recent study showed that wingless/int-1 homolog (Wnt)3a stimulates osteoblast differentiation only in cells with intrinsic osteogenic potential and not in fat cell precursors or fibroblasts [17]. Wnt3a promotes osteoblast differentiation by stimulating bone morphogenetic protein 2 (BMP2) production. In non-osteogenic cells, CpGs islands in *BMP2* and *alkaline phosphatase (ALP)* promoters show increased methylation leading to prevention of their expression. Moreover, treatment of these non-osteogenic cells with 5′-aza-2′-deoxycytidine, a CpG-demethylating agent, makes *BMP2* and *ALP* genes receptive to Wnt3a [17].

DNA methylation marks are also important for the crosstalk between osteoblasts and osteoclasts. Osteoblasts express receptor activator of nuclear factor-kappa B ligand (RANKL) on the extracellular surface of their plasma membrane, which binds to RANK, activating signaling pathways that promote osteoclast differentiation and survival. Higher levels of CpG methylation of the *RANKL* promoters were detected in various tissues expressing low or no levels of RANKL [18]. Consistently, treatment with the DNA demethylating agent 5′-aza-2′-deoxycytidine promoted a 170-fold induction of RANKL mRNA expression in HEK-293 cells, which showed hypermethylation of the CpG islands and hardly expressed RANKL transcript at baseline [18]. Conversely, treatment with DNA methyltransferase inhibitor restored RANKL expression, suggesting that CpG methylation of the *RANKL* promoter reversibly suppresses RANKL gene expression. DNA methylation affects not only the genes that activate osteoclast differentiation but also those that repress it. Recently, it has been shown that DNA methylation by DNMT3a, mediated by *S*-adenosylmethionine (SAM), regulate osteoclastogenesis by epigenetic repression of *interferon regulatory factor 8* (*IRF8*) [19]. IRF8 is a key negative regulator of osteoclast phenotype that needs to be epigenetically silenced for osteoclastogenesis to proceed. In an in vivo model, mice that lacked Dnmt3a in their osteoclasts showed higher bone mass due to a reduced number of osteoclasts, suggesting the role of DNMT3a in bone homeostasis [19].

Beside osteoclasts and osteoblasts, osteocytes represent the third and the far most abundant bone cell type. Osteocytes are considered to be osteoblasts that have reached their ultimate state of differentiation and become progressively embedded in mineralized bone. The osteoblast-to-osteocyte transition is also modulated by DNA methylation. The expression of *ALP* and *sclerostin* (*SOST*) genes is regulated by CpG methylation [20]. Indeed, hypermethylation of *ALP* and *SOST* promoters negatively correlates with gene expression in osteoblastic cells. Moreover, the methylation of those promoters changes during osteoblast-to-osteocyte transition and controls gene expression in acell-specific manner [21,22]. The number of genes important for osteoblast and osteoclast differentiation and for the osteoblast-to-osteocytes transition are regulated by DNA methylation [23,24,25]. However, other epigenetic modifications, such as chromatin modifications and ncRNA gene regulations, are also involved in the differentiation and activity of bone cells [26].

## 4. Histone Modifications

**Figure 3:**
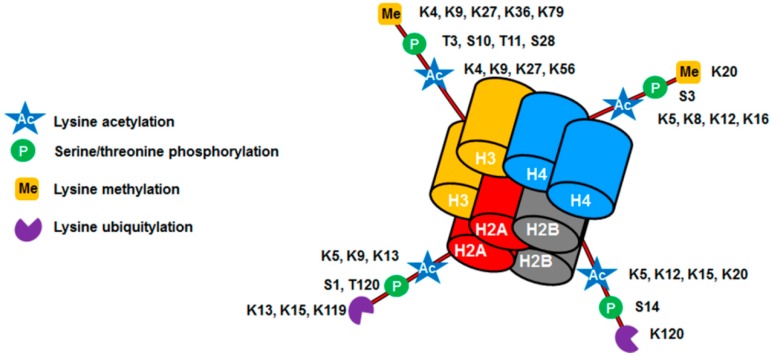
Nucleosome with histone post-translational modifications. Inside the nucleus DNA is wrapped into a protein complex known as chromatin. This protein complex (nucleosome) is composed of an octamer of four different histones (H3, H4, H2A, and H2B). Histones display a large number of modified residues (acetylation, methylation, phosphorylation and ubiquitylation). Through these modifications, chromatin becomes very dynamic, controlling the expression or repression of specific genes.

Histone modification is another epigenetic mechanism that regulates gene expression. In eukaryotic cells, DNA is complexed with histones which leads to its compaction and assembly into the basic unit of chromatin—the nucleosome. Histone modifications are key components of epigenetic regulation by which the cells regulate transcription, replication and repair [23]. Modifications occur on accessible tails and can regulate chromatin structure (Figure 3).

In addition to the well-characterized acetylation, methylation, phosphorylation and ubiquitylation modifications, recent studies have revealed other new types of histone marks such as propionylation, butyrylation, malonylation, glycosylation, etc. [27,28,29]. The main reversible histone modifications are listed in Table 1.

The histone lysine acetylation (Kac) has been widely studied and shown to be closely linked to transcriptional regulation. Early studies revealed the association of hyperacetylated histones, which results in uncompressed chromatin, with actively transcribed genes, suggesting a role for histone acetylation in gene activation by increasing the accessibility of DNA to the transcription machinery [40,41]. In contrast, histone deacetylation results in condensed chromatin, leading to gene inactivation. The addition of acetyl groups on lysine residues in histone tails implicates lysine acetyltransferases (KATs)/histone acetyltransferases (HATs), whereas their elimination involves histone deacetylases (HDACs).

In several bone candidate genes, histone modifications have been studied in terms of their effects on gene expression and cell differentiation. In murine bone stromal cells, the histone deacetylase inhibitors trichostatin A (TSA) and sodium butyrate strongly increased *RANKL* promoter activity through enhanced acetylation of histone H3 and H4 [42]. However, unlike what is expected, TSA suppressed RANKL-induced osteoclast formation from primary bone marrow-derived macrophages, suggesting that the effects of these histone modifications must be taken into account as cell-specific consequences as well as in a broader context [43].

Recently, Rojas et al. showed that the epigenetically-forced expression of *Runx2* and *osteocalcin*, classical bone-related target genes, under myoblastic differentiation is accompanied by enrichment of the H3K4me3 and H3K27ac marks at the *Runx2* promoter region [44]. These authors identified JARID1B, also known as lysine (K)-specific demethylase (KDM)5B, as a key and potent epigenetic switch which controls mesenchymal cell differentiation into myogenic and osteogenic lineages. During osteoblast differentiation, acetylation of histone H3 and H4 were significantly enhanced at the promoters of the *osterix* and *osteocalcin* genes, osteoblast markers genes, whereas histone deacetylase 1 (HDAC1) recruitment at those promoters was downregulated [45]. Moreover, knockdown of HDAC1 by the short interference RNA (siRNA) stimulated osteoblast differentiation. It was also shown in a recent study that the treatment of non-osteogenic cells with TSA allows Wnt3a to promote osteogenesis in these cells, suggesting that direct conversion of non-osteogenic cells into osteoblastic cell types without inducing pluripotency might be controlled by histone modifiers [17].

## 5. Non-Coding RNAs

Although 90% of genomic DNA is transcribed into RNA, only 1%–2% of the human genome encodes for proteins [46]. A vast majority of RNA is an end product not used to make proteins and is represented by ncRNAs. These ncRNAs play significant roles in the regulation of gene expression through transcriptional and post-transcriptional regulation [47,48]. Non-coding RNAs can be divided into two main groups: infrastructural and regulatory ncRNAs. Since they are involved in the modification and regulation of other RNAs, regulatory ncRNAs are considered as epigenetic modifiers. They can be categorized into six groups: microRNAs (miRNAs), P-element induced wimpy testis (piwi)-interacting RNAs (piRNAs), siRNAs, long non-coding RNAs (lncRNAs), enhancer RNAs (eRNAs), and promoter-associated RNAs (PARs). These ncRNAs are characterized by different lengths and different functions (Table 2).

Based on the length, ncRNAs can be divided into two groups: the short ncRNAs (<30 nts) and the long ncRNAs (lncRNAs) (>200 nts) however, both groups are shown to play a role in heterochromatin formation, histone modification, DNA methylation targeting, and gene silencing [49]. Despite the fact that lncRNAs are key regulators of diverse biological processes such as cell growth and differentiation, little is known about whether they regulate bone cell differentiation and bone homeostasis. However, a recent study has demonstrated that anti-differentiation ncRNA (ANCR) which is affiliated with the lncRNA class and more recently named differentiation antagonizing non-protein coding RNA (DANCR) is an essential mediator of osteoblast differentiation [50]. DANCR overexpression is sufficient to inhibit osteoblast differentiation and DANCR-siRNA promotes osteoblast differentiation. More recently, Tong et al. showed that the expression of DANCR is upregulated in blood mononuclear cells from low bone mineral density (BMD) patients. DANCR promotes the expression of interleukine (IL)-6 and tumor necrosis factor (TNF)-α, and the level of DANCR was correlated with IL-6 and TNF-α in postmenopausal women [51].

The most studied ncRNAs in relation to bone homeostasis and bone diseases are miRNAs. In the last decade a large number of miRNAs were clearly and closely associated to the development and metabolism of bone. Additionally, the altered expression levels of some miRNAs can cause bone metabolism disorders leading to osteoporosis [52,53,54]. Many studies have reported that the expression of certain miRNAs in osteoblast and osteoclast differentiation appears upregulated or downregulated [55,56,57,58,59,60]. Among all described miRNAs, there are two groups: miRNAs promoting differentiation and miRNAs inhibiting differentiation of bone cells (Table 3A).

In bone homeostasis preservation, osteoblast differentiation is an important process. Osteoblast lineage commitment is tightly regulated at both transcriptional and post-transcriptional levels. Runx2, a master regulator of osteoblast differentiation, controls the expression of bone-related genes. Transcriptional regulation of *Runx2* gene in mesenchymal progenitor cells is controlled by several miRNAs. For instance, during adipocyte differentiation miR-204 expression is induced, whereas Runx2 protein expression is suppressed, suggesting that miR-204 acts as an inhibitor of *Runx2* [61]. The involvement of miRNAs in osteoblast differentiation can also be indirect. In this context, it was recently shown that miR-15b promotes osteoblast differentiation by protecting Runx2 protein from mothers against decapentaplegic homolog (SMAD) specific E3 ubiquitin protein ligase 1 (Smurf1) mediated degradation [62]. Compared to osteoblast differentiation, the impact of miRNAs on osteoclast differentiation has been less studied [60,61,62,63] (Table 3B).

Nonetheless, the expression pattern of miRNAs during the osteoclast differentiation has been explored and has shown changes in several miRNAS [78,79]. Recently the functions of relevant miRNAs in osteoclasts and related bone diseases, such as osteoporosis, have been summarized [80]. Osteoclasts are derived from mononuclear macrophages and the most important step in osteoclast differentiation is the fusion of mononucleated cells to form multinucleated cells. Dendritic cells-specific transmembrane protein (DCSTAMP) is a key regulator of osteoclast cell fusion and differentiation [81,82]. By targeting DCSTAMP, miR-7b inhibited osteoclastogenesis and cell fusion. The inhibition of DCSTAMP influences the expression of other genes involved in osteoclast fusion and differentiation such as nuclear factor of activated T-cells 1 (NFATC1), C-Fos, protein kinase B/serine-threonine protein kinase (AKT) and TNF receptor associated factor 6 (TRAF6) [75]. Recently it was shown that connective tissue growth factor/CCN family 2 (CTGF/CCN2), which can promote osteoclast formation via upregulation of DCSTAMP is targeted by miR-26a [74]. The overexpression of miR-26a inhibitor enhanced RANKL-induced osteoclast formation and function as well as CTGF expression, suggesting that miR-26a modulates osteoclast formation and function through the regulation of CTGF.

## 6. Epigenetic and Osteoporosis

Osteoporosis is a skeletal disorder characterized by a reduction of bone strength and increased risk of bone fracture. Many factors influence bone homeostasis and bone mineral accumulation including heredity, gender, diet, physical activity, endocrine status, and sporadic risk factors such as smoking. In addition to these modifiable factors during childhood, evidence has also accrued that fracture risk might be programmed during intrauterine life. Lately, the involvement of epigenetic gene regulation in many diseases has been documented making it an important target for basic and clinical research for multifactorial diseases including osteoporosis.

The number of studies suggesting that epigenetic modifications play an important role in bone homeostasis and BMD has become increasingly significant [83,84,85]. The osteoprotegerin (OPG)/RANKL system is important in the equilibrium between bone formation and bone resorption. The alteration of this system is also involved in bone loss that characterizes osteoporotic patients [86]. The association between OPG:RANKL ratio and DNA methylation has recently been proposed to explain, at least partially, osteoclast activation and osteoporotic fractures [18]. It is well established that estrogen deficiency induces osteoporosis. Methylation of the promoter A region in the *estrogen receptor alpha* (*ERα*) gene, which leads to a decrease ERα mRNA, is increased in post-menopausal women than pre-menopausal women [87]. Thus, DNA methylation seems to be an important mechanism for the pathogenesis of osteoporosis. However, other mechanisms appear to be involved in the emergence of osteoporosis. Indeed, histone modifications impact bone cell differentiation and ultimately the onset of osteoporosis. During osteoporosis, the imbalance between bone mass and fat increases the risk of fracture. The histone demethylases KDM4B and KDM6B play critical roles in osteogenic commitment of MSCs by removing methyl group from H3K9 and H3K27. Depletion of KDM4B or KDM6B significantly reduces osteogenic differentiation and increases adipogenic differentiation [88]. In ovariectomized mice H3K27me and H3K9me-positive MSCs were significantly elevated suggesting a link between histone modifications and osteoporosis. Other studies showed the impact of histone modifications on MSC differentiation and their implication in bone fracture risk. On the promoters of *Wnts* (Wnt1, Wnt6 and Wnt10) histone methylation (H3K27me) by the enhancer of zeste homolog 2 (EZH2) inhibits Wnt genes transcription. This Wnt/β-catenin pathway inhibition is responsible of the shift of MSC lineage commitment towards adipocytes [89]. The shift of MSCs towards adipogenic differentiation is also controlled by HDACs. Indeed, the suppression of HDAC3 activity contributes to increase marrow adiposity associated with aging bone and osteoporosis [90]. Given that histones are chemically modifiable, handling their activities represents a potential and promising way to treat bone diseases such as osteoporosis.

There is accumulating evidence that miRNAs play a critical role in the regulation of various biological processes including bone homeostasis. MicroRNAs have been deeply involved in the regulation of bone cells differentiation and bone resorption. Recent studies demonstrated that the expression of several miRNAs is markedly upregulated in the serum of patients with osteoporotic fractures and can influence osteogenic differentiation [54,91]. Since miRNAs are the most abundant RNA species to be found in circulation, quantification of their expression may be used as biomarker for diagnostic purposes and may be a target for drug development [92,93,94]. Compounds targeting specific miRNAs are currently in clinical trials for the treatment of cancer [95] or type II diabetes [96]. In a recent study, resveratrol, which is a polyphenolic phytoestrogen with osteogenic and osteoinductive properties, exhibited the ability to prevent osteoporosis by suppressing miR-338-3p. Mechanistically, resveratrol treatment leads to suppressed miR-338-3p, followed by an increase in Runx2 expression [97].

## 7. Epigenetically Active Drugs and Bone Homeostasis

In the last few decades, HDAC inhibitors and bromodomain (BRD) inhibitors were developed to serve as anticancer drugs via inhibiting the deacetylation of histones or the recognition of acetyl-lysine groups on histones or non-histone proteins respectively [98,99]. For bone homeostasis the regulation of *SOST* expression was found to be an important target of HDACs, since SOST inhibits bone formation by antagonizing canonical Wnt signaling, which is required for normal osteoblastogenesis and control of osteoclastogenesis [100]. *SOST* gene expression was found to be negatively regulated by HDAC5 by inhibiting myocyte enhancer factor 2 (Mef2) and positively regulated by class I HDACs [100]. The bone anabolic effect of the HDAC I inhibitor MS-275, however, was also found to derive from interference with the DExH-box helicase Dhx36 [101]. In essence, class I HDAC inhibitors represent a novel approach for bone forming osteoporosis therapies [100].

Inhibitors of the BRD target osteoblastogenesis via *Runx2* inhibition and osteoclastogenesis via inhibition of Myc and nuclear factor kappa B (NFκB) [102]. In the context of the treatment of osteoporosis and bone loss, BRD inhibitors have shown in vitro and in vivo promising results presumably due to their activity to inhibit osteoclastogenesis and inflammation [98,102,103,104,105,106,107]. Indeed, DNA methylation and histone modifications such as acetylation could be recognized by BRD-containing proteins and selective BRD inhibitors seem to be a new epigenetic approach to treat bone-related diseases. For example, the BRD inhibitor I-BET151 targets an epigenetic mechanism important for osteoclast differentiation and demonstrates therapeutic efficacy by preserving bone mass and strength in an ovariectomy-based model of osteoporosis [108]. In light of the strong inhibition of *Runx2* expression, however, BRD inhibitors might not be ideally suited for osteoporosis treatment, since the transcription factor Runx2 is needed for the onset of osteoblast differentiation from MSCs [109]. In contrast to high affinity BRD inhibitors like JQ1 or I-BET151, low affinity BRD inhibitors like *N*-methyl pyrrolidone (NMP) were shown to facilitate differentiation of osteoblasts from human MSCs, enhance bone regeneration and bone formation [110]. During the last few years, we demonstrated that NMP stimulates bone formation and inhibits osteoclast differentiation and bone resorption [103,110]. Furthermore, we established that this small bioactive molecule acts as a BRD inhibitor and is able to inhibit inflammatory mediators and to prevent bone loss in an animal model of osteoporosis [104,105]. In essence, the low affinity BRD inhibitor NMP is suited for osteoporosis treatment since inflammation and bone degradation are reduced and at the same time bone formation enhanced.

## 8. Conclusions

This review illustrates that epigenetic regulation is deeply involved in bone homeostasis and that epigenetic-based therapeutics have already shown potential for the treatment of osteoporosis. Epigenetically active drugs like HDACS and BRD inhibitors target several genes and pathways, therefore further studies are required to translate these findings to clinical trials and later to patients.

## Figures and Tables

**Table 1:** The main reversible histone modifications.

Enzyme	Target	Modification	References
Histone acetyltransferases (HATs), Histone deacetylases (HDACs)	Lysine	Acetylation Deacetylation	[30,31,32,33]
Lysine methyltransferases, arginine methyltransferases	Lysine Arginine	Methylation Demethylation	[34,35,36]
Kinases, phosphatase	Serine Threonine Tyrosine	Phosphorylation Dephosphorylation	[37]
Ubiquitin ligase (E3) and ubiquitin-activating enzyme (E1/E2), Small Ubiquitin-like Modifier (SUMO)	Lysine	Ubiquitylation Sumoylation Deubiquitylation	[38]
Poly (ADP-ribose) polymerase (PARP)	Glutamate Arginine	ADP-ribosylation	[38,39]

**Table 2:** Non-coding RNA (ncRNA) and their characteristics and functions.

Name	Length (nt)	Characteristic and Function
MicroRNA (miRNA)	20–24	● Single-stranded RNA (ssRNA) ● derived from pre-miRNA (hairpin) ● gene silencing
Piwi-interacting RNA (piRNA)	24–31	● Form complexes with P-element induced wimpy testis (Piwi) proteins of the Argonaute family ● silencing of transposable elements
Small interfering RNA (siRNA)	20–24	● Double-stranded RNA (dsRNA) processed by endoribonuclease Dicer into mature siRNA ● post-transcriptional silencing/RNA interference (RNAi) ● protection against viral infection
Promoter-associated RNA (PAR)	16–200	● ssRNA with short half-life ● Post-transcriptional regulation
Enhancer RNA (eRNA)	100–9000	● ssRNA with short half-life ● trasncriptional gene activation
Long non-coding RNA (lncRNA)	>200	● Non-protein coding transcripts ● Subject to post-transcriptional modifications ● Transcriptional/post-transcriptional regulation and precursor for siRNA

**Table 3:** Example of functional miRNAs in osteoblast (A) and osteoclast (B) differentiation.

miRNAs	Target	Effect	References
**A. Osteoblast Differentiation**			
miR-216-a	PI3K/AKT pathway	E	[64]
miR-21	SMAD7	E	[65]
miR-194	STAT1	E	[66]
miR-96	EGFR signaling	E	[67]
miR-23-a	GjA1	I	[68]
miR-375	Runx2	I	[69]
miR-153	BMPRII	I	[70]
miR-124	Dlx5, Dlx3, Dlx2	I	[71]
**B. Osteoclast Differentiation**			
miR-214	PTEN/AKT pathway	E	[60]
miR-183	Heme oxygenase-1	E	[72]
miR-9718	PIAS3	E	[73]
miR-17/20a	RANKL	I	[59]
miR-26-a	CTGF	I	[74]
miR-7-b	DCSTAMP	I	[75]
miR-34-a	TGIF2	I	[76]
miR-126-5p	MMP13	I	[77]

E, enhances; I, inhibits. AKT, protein kinase B/serine-threonine protein kinase; BMPRII, bone morphogenetic protein receptor type II; CTGF, connective tissue growth factor; DCSTAMP, dendritic cells-specific transmembrane protein; Dlx, distal-less homeobox; EGFR, epidermal growth factor receptor; GjA1, gap junction protein alpha 1; MMP, matrix metallopeptidase; SMAD7, mothers against decapentaplegic homolog 7; PTEN, phosphatase and tensin homolog; PIAS3, protein inhibitor of activated STAT 3; PI3K, phosphatidylinositol-4,5-bisphosphate 3-kinase; RANKL, receptor activator of nuclear factor-kappa B ligand; Runx2, runt related transcription factor 2; STAT1, signal transducer and activator of transcription 1; TGIF2, TGFB induced factor homeobox 2.
